# Recent Advances in Triboelectric Nanogenerators for Biomedical and Cardiovascular Monitoring

**DOI:** 10.3390/ma19081647

**Published:** 2026-04-20

**Authors:** Amit Sarode, Jegan Rajendran, Gymama Slaughter

**Affiliations:** 1 Center for Bioelectronics, Old Dominion University, Norfolk, VA 23508, USA; 2 Department of Engineering, Norfolk State University, Norfolk, VA 23504, USA; 3 Department of Electrical and Computer Engineering, Lawrence Technological University, Southfield, MI 48075, USA; 4 Department of Electrical and Computer Engineering, Old Dominion University, Norfolk, VA 23508, USA

**Keywords:** triboelectric nanogenerator, self-powered biosensing, wearable sensors, implantable sensors, cardiovascular monitoring, cuffless blood pressure, machine learning in diagnostics

## Abstract

Triboelectric nanogenerators (TENGs) have emerged as versatile self-powered platforms for wearable and implantable biomedical sensing, offering an alternative to battery-dependent electronic devices. By converting biomechanical energy from physiological motion into electrical signals, TENGs enable simultaneous energy harvesting and active sensing within flexible, lightweight, and biocompatible architectures. This review summarizes recent advances from 2020 to 2025 in triboelectric nanogenerator (TENG)-based cardiovascular monitoring. The discussion focuses on material systems, device configurations, sensing mechanisms, and applications including pulse detection and cuffless blood pressure estimation. Representative studies are compared to highlight emerging trends in wearable and self-powered sensing technologies. However, differences in experimental conditions, anatomical sites, calibration methods, and signal-processing approaches limit direct comparison of reported performance. In addition, challenges such as subject-specific calibration, motion artifacts, and limited clinical validation remain. Overall, this review highlights current progress and outlines key challenges for future development and translation of TENG-based cardiovascular monitoring systems.

## 1. Introduction

The increasing global burden of chronic and acute diseases has accelerated the demand for continuous, noninvasive biomedical monitoring capable of capturing dynamic physiological changes in real time rather than through intermittent, clinic-based measurements. Wearable and noninvasive technologies enable continuous tracking of vital signs including heart rate, blood oxygen saturation, body temperature, and other biomarkers facilitating early identification of disease onset or exacerbation [1]. Such longitudinal data acquisition has become central to personalized healthcare and preventive medicine, empowering both patients and clinicians with real-time physiological insight beyond clinical environments. Recent reviews have emphasized the transformative impact of flexible sensing platforms and continuous physiological data streams in remote health assessment and chronic disease management [2].

Despite this progress, traditional biomedical sensors remain constrained by reliance on external power supplies, limited wearability, mechanical rigidity, and user discomfort, which collectively hinder uninterrupted real-world operation [3,4,5]. These limitations have driven the exploration of self-powered sensing systems capable of autonomous and seamless integration with the human body [6]. Among various energy-harvesting technologies, including piezoelectric nanogenerators (PENGs), triboelectric nanogenerators (TENGs) have emerged as particularly promising due to their high sensitivity, structural flexibility, lightweight configuration, and biocompatibility [7,8,9].

**Figure 1:**
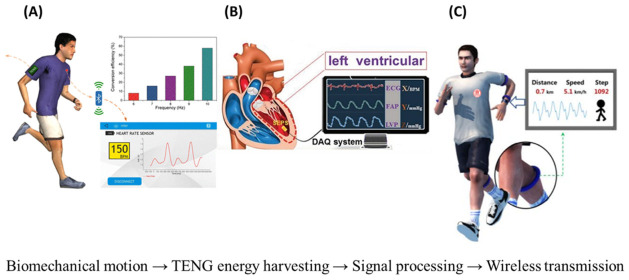
Schematic overview of triboelectric nanogenerators (TENG)-based biomedical systems: (**A**) wearable sensing for motion and respiration, (**B**) cardiovascular monitoring (ECG, pulse waveform, blood pressure), and (**C**) self-powered platforms integrating energy harvesting, signal processing, and wireless transmission, illustrating a unified framework for wearable and intelligent biomedical sensing [8,16,17].

Unlike battery-dependent platforms, TENGs convert biomechanical energy from natural human motion and physiological processes directly into electrical signals, enabling simultaneous energy harvesting and active sensing within a single device architecture [10]. These features make TENGs especially suitable for wearable and implantable biomedical applications, where continuous, noninvasive, and user-friendly operation is essential [11,12]. Recent studies have demonstrated TENG-based systems for cardiovascular, respiratory, and motion monitoring, as well as their integration into smart textiles and electronic skin platforms [13,14]. This self-powered capability, combined with mechanical flexibility and material adaptability, supports their integration into wearable and implantable sensing platforms for real-time physiological monitoring [15]. Figure 1 illustrates a unified framework of TENG-based biomedical systems, integrating energy harvesting, self-powered sensing, signal processing, and wireless data transmission. As shown, these platforms enable continuous monitoring of motion, respiration, and cardiovascular signals, including ECG, pulse waveforms, and blood pressure.

Although significant advances have been reported, challenges remain in achieving long-term signal stability, reproducibility, biocompatibility, environmental robustness, and scalable manufacturing for clinical translation. Integration with wireless communication, multimodal sensing, and intelligent data-processing frameworks also remains an evolving area. Previous reviews have addressed specific subsets of TENG-based biomedical applications, including wearable motion sensors, respiration-driven platforms, and biodegradable implantable systems. However, a unified critical synthesis integrating cardiovascular monitoring and hybrid intelligent TENG-enabled systems over the 2020–2025 period remains lacking. In particular, the relationship between materials innovation, device architecture, and application-level performance has not been systematically evaluated. To address this gap, this review provides a structured analysis of recent advances and identifies key translational bottlenecks toward clinically viable, multifunctional, self-powered sensing systems. Unlike prior application-specific perspectives, this work emphasizes the convergence of wearable sensing, cardiovascular monitoring, and intelligent system integration within a unified framework.

## 2. Fundamental Principles and Operating Modes of TENG-Based Biomedical Systems

### 2.1. Fundamental Mechanism: Triboelectric Effect and Electrostatic Induction

The operation of TENGs is based on the coupling of triboelectrification (contact electrification) and electrostatic induction [13,15,18]. When two materials with different electron affinities come into contact and are subsequently separated, interfacial charge transfer occurs, resulting in oppositely charged surfaces [19,20]. This triboelectric effect is a universal phenomenon observed across polymers, metals, textiles, and biological substrates, enabling broad adaptability for biomedical interfaces. Upon separation, the resulting charge imbalance generates a potential difference between the surfaces. Through electrostatic induction, electrons flow through an external circuit to balance this potential, producing an alternating electrical output [21]. The periodic coupling of charge generation and induced electron flow defines the fundamental working mechanism of TENG systems [8,22].

**Figure 2:**
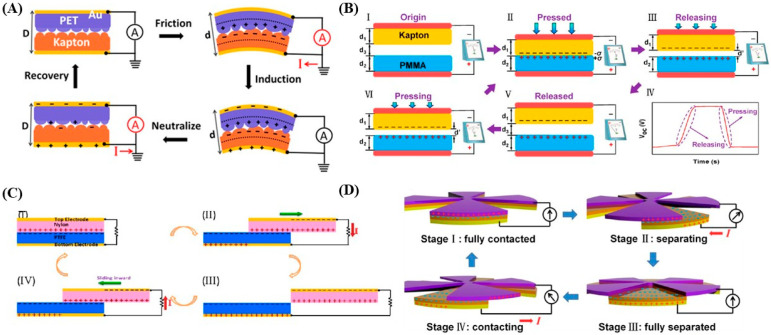
Fundamental working mechanism and representative architectures of TENGs, illustrating (**A**) contact electrification, (**B**) electrostatic induction, and the primary operating modes including (**C**) contact–separation, sliding, and (**D**) rotational configurations [13,15,18].

The electrical output of TENGs is governed by several interdependent parameters, including surface charge density, effective contact area, dielectric constant, surface micro/nanostructure, separation distance, and mechanical deformation frequency. In biomedical applications, these parameters must be optimized not only for electrical performance but also for mechanical compliance, biocompatibility, and long-term stability. Accordingly, surface engineering strategies, such as microstructured elastomers, high-dielectric composite films, and flexible polymer interfaces, have been widely explored to enhance charge density and signal output while maintaining durability under repetitive biomechanical loading [23]. These approaches highlight the versatility of TENG architectures across wearable and implantable platforms, supporting their dual role as both power sources and sensing transducers. As illustrated in Figure 2, TENG operation follows a cyclic process of contact, separation, and charge redistribution across distinct configurations, including contact-separation, lateral sliding, and rotational modes. Despite differences in geometry and actuation, all configurations share a common mechanism based on triboelectric charge formation and electrostatic induction-driven electron flow. This structural diversity enables efficient conversion of various biomechanical motions into electrical signals, facilitating broad implementation in wearable and implantable biomedical systems.

### 2.2. Operating Modes for Biomedical Sensing

**Figure 3:**
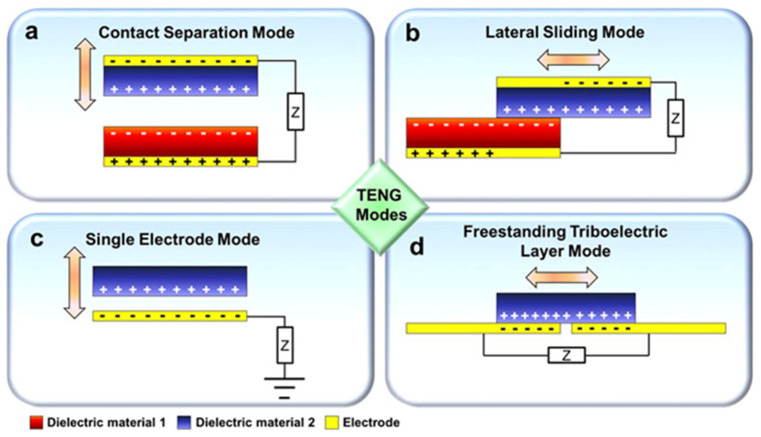
Schematic illustration of the four fundamental TENG operating modes: (**a**) contact–separation, (**b**) lateral sliding, (**c**) single-electrode, and (**d**) freestanding triboelectric layer. Electrical output arises from periodic modulation of electrostatic potential induced by mechanical motion between triboelectric materials [28].

Each *TENG* configuration exhibits distinct characteristics in sensitivity, mechanical adaptability, and suitability for specific physiological signals [18,24]. The contact–separation mode is one of the most widely utilized configurations for biomedical sensing due to its structural simplicity and high output performance [25,26]. In this mode, two triboelectric layers periodically contact and separate under external mechanical stimuli such as respiration, gait, joint motion, or arterial pulsation [13]. These cyclic interactions generate alternating charge flow, enabling sensitive detection of subtle biomechanical variations with high temporal resolution [27]. As illustrated in Figure 3a, vertical deformation modulates the effective contact area between triboelectric layers, thereby regulating the induced potential and output amplitude. This configuration is particularly suitable for monitoring pulse waveforms, plantar pressure, and thoracic expansion, where normal compressive forces dominate. Despite its high sensitivity and output (typically tens to several hundred volts, with power densities up to 500 mW m^−2^ [27]. This mode may experience mechanical fatigue under repetitive loading and requires effective encapsulation for stable operation in moist or in vivo environments.

In the lateral sliding mode, relative tangential displacement between triboelectric surfaces induces dynamic charge redistribution [28]. Compared with contact-separation mode, sliding-mode TENGs are more responsive to shear forces and multidirectional motion [29], making them well-suited for joint tracking, rehabilitation monitoring, and wearable motion analysis. However, friction-induced wear and long-term durability remain key limitations. Typical output voltages range from 10 to 500 V with moderate power density [28,29].

In addition, the single-electrode mode simplifies device architecture by employing one active triboelectric layer, with the environment or human body serving as the reference ground [25]. This eliminates the need for paired layers, enabling straightforward integration onto skin, textiles, and implantable substrates. As a result, this configuration is particularly attractive for wearable and epidermal sensing applications. However, output performance (typically 1–100 V) is lower and can be influenced by environmental grounding conditions, requiring careful calibration [25].

Whereas, in the freestanding triboelectric layer mode, a charged dielectric moves between two fixed electrodes without direct simultaneous contact [30]. This configuration enhances charge transfer efficiency while reducing mechanical wear, making it advantageous for enclosed or implantable systems. However, increased structural complexity and fabrication precision may limit scalability. Output performance is comparable to contact–separation configurations but depends strongly on alignment and device design.

### 2.3. Comparative Considerations for Biomedical Translation

Among the four operating modes, contact-separation and single-electrode configurations are most widely used in biomedical sensing due to their compatibility with flexible substrates, ease of fabrication, and strong signal output [27]. A defining feature of all TENG systems is their dual functionality as both energy harvesters and active sensors [31]. This capability distinguishes TENGs from conventional battery-powered biosensors and supports their use in autonomous wearable and implantable devices. To enable quantitative comparison, Table 1 summarizes key performance metrics, including output voltage, power density, sensitivity, structural complexity, and application suitability. Contact-separation mode typically provides the highest voltage output and pressure sensitivity, while lateral sliding mode offers superior response to shear motion. Single-electrode configurations simplify device integration but are influenced by environmental grounding conditions. In contrast, freestanding triboelectric-layer mode enhances charge transfer efficiency but requires greater fabrication precision.

Overall, these trade-offs highlight the importance of selecting operating modes based on the specific mechanical stimuli, integration requirements, and clinical application context.

## 3. Materials Innovations Driving TENG-Based Biomedical Systems

**Figure 4:**
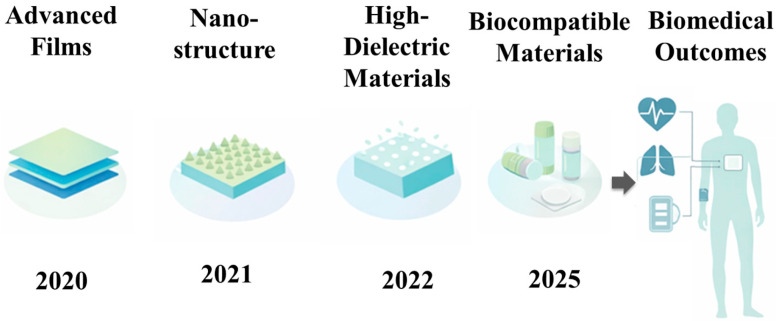
Material and structural evolution of triboelectric nanogenerators (2020–2025), highlighting advances in triboelectric polymers (e.g., PDMS, PTFE) [33,34,35], micro/nanostructuring strategies [36], high-dielectric hydrogels [37,38,39], and biodegradable/bioresorbable materials [40] for high-performance self-powered biomedical sensing.

From 2020 to 2025, advances in triboelectric polymers, micro/nanostructured interfaces, high-dielectric soft materials, and biodegradable platforms have significantly improved the feasibility of wearable and implantable self-powered systems. Similar to how receptor chemistry governs performance in molecularly imprinted polymer systems [32], the electrical output, sensitivity, mechanical compliance, and long-term stability of TENG devices are dictated by triboelectric material selection, surface architecture, dielectric enhancement, and biocompatibility. Figure 4 summarizes these key material and structural developments.

Conventional polymers including polydimethylsiloxane (PDMS), polytetrafluoroethylene (PTFE), and fluorinated copolymers continue to dominate biomedical TENG design due to their strong electron affinity, chemical stability, and mechanical flexibility. PDMS, a soft and stretchable elastomer, is widely employed in wearable and implantable systems because of its ability to conform to curved and dynamic biological surfaces [33]. Its low Young’s modulus enables stable signal generation under repetitive deformation, making it particularly suitable for pulse, respiration, and joint motion sensing. However, PDMS inherently exhibits moderate dielectric properties and may require surface modification to improve charge retention. PTFE and related fluoropolymers exhibit superior negative triboelectric polarity and enhanced charge trapping capability. These materials are frequently integrated into high-output TENG architectures designed for motion sensing and energy harvesting [34]. Recent developments in fluorinated copolymers and surface fluorination treatments have further increased dielectric constant and surface charge density, resulting in improved voltage amplitude and output stability suitable for biomedical transduction platforms [35].

Beyond intrinsic material properties, surface morphology plays a critical role in triboelectric performance. Increasing effective contact area and local electric field intensity enhances charge density and signal amplitude. Accordingly, micro- and nanostructuring has become a key strategy for performance enhancement in TENG-based biomedical sensors. Structured architectures incorporating carbon nanotube (CNT) pillars, nanoporous frameworks, graphene coatings, and composite microtextures have been widely explored. For example, CNT-PDMS composites with vertically aligned pillars exhibit enhanced current density and improved signal-to-noise ratios in wearable motion and pulse monitoring systems. The incorporation of conductive nanomaterials promotes localized electric field concentration and facilitates interfacial charge trapping.

In addition, polyvinylidene fluoride (PVDF), a semi-crystalline polymer exhibiting both triboelectric and piezoelectric properties, has been hybridized with graphene and dielectric nanoparticles to form synergistic triboelectric-piezoelectric systems [36]. This dual-mode coupling enhances sensitivity and broadens the dynamic response range, particularly in applications involving multi-directional strain and pressure. Micro/nanostructured surfaces also improve mechanical durability and cyclic stability, which are essential for long-term wearable operation.

Complementing surface engineering approaches, the integration of high-dielectric gels and ionic hydrogels has enabled a shift toward soft, skin-conformal, and implant-compatible TENG platforms. Hydrogels based on polyacrylamide, polyvinyl alcohol, and ionic liquids exhibit intrinsic ionic conductivity and high mechanical compliance, enabling intimate coupling with biological tissues. These materials can function as both triboelectric layers and stretchable electrodes, reducing interfacial impedance and improving signal transduction [37].

Incorporation of high-dielectric nanoparticles such as BaTiO_3_ and TiO_2_ further enhances surface polarization and charge accumulation, resulting in increased power output and sensitivity [38,39]. Hydrogel-based TENGs (H-TENGs) demonstrate exceptional flexibility and stretchability, supporting stable operation under complex body motion. Their soft mechanical properties reduce interfacial mismatch and improve comfort during prolonged wear. However, challenges including dehydration, mechanical degradation, and long-term ionic stability must be addressed to ensure reliable operation in physiological environments.

In parallel, the development of biodegradable and bioresorbable materials is advancing sustainable and transient biomedical electronics. Materials such as polylactic acid (PLA), silk fibroin, and gelatin are increasingly incorporated into TENG architectures to enhance environmental sustainability and biosafety. Silk-based TENGs offer a favorable combination of mechanical strength, flexibility, and biocompatibility, making them suitable for both epidermal and implantable interfaces. PLA provides an eco-friendly substrate for disposable or short-term sensors, while gelatin-based composites combine softness with biodegradability and improved triboelectric performance [40]. Therefore, bioresorbable TENGs based on these materials have demonstrated transient energy harvesting capabilities, enabling temporary powering of implantable devices without the need for surgical removal.

Across these material categories, several overarching trends define the evolution of TENG-based biomedical systems. There has been a clear transition from rigid polymer films toward soft, stretchable, and skin-conformal materials that better match the mechanical properties of biological tissues, improving comfort, signal stability, and long-term wearability. In parallel, micro- and nanostructuring strategies have significantly enhanced charge density, local electric field concentration, and sensing sensitivity. The incorporation of dielectric-enhancing nanoparticles within polymer and hydrogel matrices has further improved electrical output, enabling higher voltage amplitudes and reliable signal generation under low-amplitude biomechanical stimuli.

At the same time, increasing emphasis on biocompatibility, biodegradability, and environmental sustainability reflects a broader shift toward implantable systems and eco-conscious disposable devices. Despite these advances, key barriers to clinical translation remain, including device reproducibility, environmental robustness under physiological conditions, and scalable manufacturing. Table 2 provides a comparative summary of material properties, performance metrics, and application relevance across major triboelectric material classes (2020–2025). This comparison highlights the trade-offs that govern material selection in biomedical TENG systems.

As shown in Table 2, fluorinated polymers deliver superior electrical output due to strong electron affinity, whereas hydrogels and biodegradable materials offer enhanced biocompatibility and mechanical compliance. These differences emphasize a fundamental trade-off between electrical performance and biological compatibility in the design of TENG-based biomedical devices. Optimizing this balance between electrical performance and biological compatibility remains a central challenge in advancing TENG materials toward clinically deployable biomedical systems.

## 4. Device Engineering Trends in TENG-Based Biomedical Systems

Building on material innovations, recent advances emphasize thin-film, stretchable, textile-integrated, and miniaturized implantable architectures optimized for continuous physiological monitoring. These developments have been critical in transitioning TENGs from proof-of-concept systems to wearable and implantable biomedical platforms. For example, thin-film TENGs fabricated from elastomeric polymers such as PDMS and polyurethane, enable efficient transduction of subtle biomechanical signals, including arterial pulsation, respiration, and muscle contraction, into electrical output [25], wherein mechanical compliance is critical for maintaining stable signal generation under bending, twisting, and tensile deformation during long-term wear.

To address this, recent designs incorporate multilayered and sandwich-type architectures that enhance charge density while improving structural durability. For example, ridged elastomeric structures and vertically compressible layers enable improved contact modulation and sustained performance under repetitive loading. These configurations function as self-powered epidermal patches capable of real-time cardiac and respiratory monitoring without external batteries. Engineered roughness at the micro- and nanoscale achieved through nanopillars, sponge-like frameworks, laser etching, or soft lithography significantly increases effective contact area and promotes charge accumulation. In addition, these porous elastomer-based TENGs provide lightweight, breathable platforms suitable for prolonged skin attachment. These micro-patterned architectures further enable tuning of pressure sensitivity, supporting detection of arterial pulse waves, vocal cord vibration, and tactile stimuli. Importantly, these structures improve signal stability and reduce mechanical wear, thereby extending device lifetime during continuous operation [33].

Furthermore, the integration of TENG functionality into textiles represents a key advancement toward scalable and user-friendly biomedical monitoring. Textile-based TENGs (T-TENGs) utilize conductive fibers, triboelectric yarns, and knitted architectures to harvest biomechanical energy from natural body motion while preserving comfort and breathability [41]. Unlike rigid sensor modules, textile-integrated systems distribute sensing and energy harvesting across large areas, improving signal robustness and reducing localized mechanical strain. Recent developments have demonstrated washable, flexible garments incorporating triboelectric fibers capable of detecting limb motion, posture variation, and respiratory expansion. The integration of conductive yarns with triboelectric fibers enables continuous physiological monitoring during daily activities, while coupling with wireless transmission modules supports real-time data streaming to IoT-enabled healthcare platforms [41].

Extending beyond wearable textiles, recent efforts have focused on miniaturized and implantable TENG systems capable of harvesting in vivo biomechanical energy. Significant progress has been made in developing soft, biocompatible, and bioresorbable architectures suitable for implantation [41]. Miniaturized TENGs fabricated from biodegradable substrates such as PLA and silk fibroin can harvest energy from organ motion, blood flow, or respiration, providing sustainable power for implantable biosensors and therapeutic systems [34]. These systems have been explored for powering pacemakers, neural stimulators, and tissue regeneration platforms. In addition, ultrasound-driven and respiration-powered TENGs enable wireless energy conversion from internal physiological stimuli, expanding the scope of implantable applications [41]. Such approaches demonstrate the feasibility of converting physiological motion directly into electrical signals for both monitoring and therapeutic actuation.

Despite these advances, key barriers to clinical translation remain, including fabrication reproducibility, mechanical reliability, environmental stability, and integration with signal conditioning and wireless communication systems. Bridging the gap between prototype performance and standardized biomedical devices represents the next critical phase in the evolution of TENG-based healthcare technologies.

## 5. Biomedical Sensing Applications of TENG-Based Systems

TENGs’ high sensitivity to subtle biomechanical stimuli and compatibility with flexible, biocompatible materials make them well suited for wearable applications [42,43,44,45]. Recent advances demonstrate that triboelectric nanogenerators (TENGs) have evolved into versatile platforms for self-powered biomedical sensing, enabling detection of biomechanical and biochemical signals with high sensitivity and flexibility [8,9,25,46]. These systems have been widely explored for motion tracking, respiratory monitoring, neuromuscular sensing, and biochemical diagnostics, with increasing emphasis on wearable integration and real-time health monitoring [43,45].

### 5.1. Physical Sensing Applications

TENG-based biomedical sensing systems can be broadly categorized into physical sensing and biochemical sensing applications. Physical sensing includes monitoring of motion, respiration, and muscle activity, where mechanical deformation is directly converted into electrical signals. These systems have gained significant attention due to their flexibility, self-powered operation, and suitability for wearable healthcare applications.

**Figure 5:**
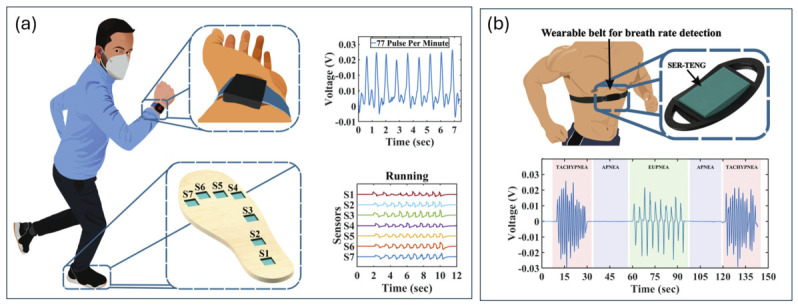
Real-time sensing using a SER-TENG device: (**a**) configuration for gait analysis and (**b**) configuration for respiration monitoring [27].

Flexible devices based on PDMS, PTFE, and nanocomposite materials enable detection of joint flexion, gait dynamics, and finger articulation, supporting applications in rehabilitation monitoring, prosthetic control, and human–machine interfaces. A representative example is a SER-TENG device developed for broadband biomechanical sensing, capable of detecting both low-amplitude physiological signals and high-force gait motions (Figure 5). The device consists of a TPU layer positioned between two textured silicone elastomer layers. Optimization of ridge geometry enhances frictional contact and separation distance, increasing charge density and electrical output. The system achieved a peak power density of 490 mW m^−2^ and a current density of 1750 µA m^−2^, while maintaining stable performance over 10,000 cycles. Demonstrations included respiration monitoring and gait analysis, highlighting its versatility for self-powered sensing.

Recent studies demonstrate that TENG-based motion sensing systems can be broadly categorized into textile-based, hydrogel-based, and hybrid nanocomposite platforms [11,25,43,46,47,48]. Textile-integrated TENGs enable continuous monitoring of joint motion and gait dynamics due to their flexibility and breathability [11,48]. Hydrogel-based systems provide enhanced stretchability and mechanical compliance, enabling detection of subtle strain signals such as muscle deformation and soft tissue motion [25,46]. Hybrid triboelectric-piezoelectric systems, including PVDF-based nanocomposites, further improve sensitivity, output performance, and durability under cyclic loading [43,46].

The performance of these systems varies depending on material selection and device architecture, with reported output voltages typically ranging from 10 to 500 V and sufficient sensitivity for detecting both subtle physiological signals and large-scale body motion. In addition, textile-integrated TENGs embedded in garments and footwear enable simultaneous energy harvesting and real-time motion tracking during daily activities [47]. These systems support continuous, battery-free monitoring while serving as auxiliary power sources for wearable electronics. These results demonstrate the capability of TENG-based systems to capture multi-scale biomechanical signals, from subtle physiological activity to high-force locomotion. As shown in Table 3, textile-based and hydrogel-based systems offer superior flexibility and conformability for wearable applications, while hybrid TENG/PENG and nanocomposite systems achieve higher output performance and sensitivity. However, these performance gains are often accompanied by increased system complexity and fabrication challenges. This comparison highlights the trade-offs between mechanical adaptability, electrical output, and long-term stability in TENG-based physical sensing systems. Despite these advances, challenges remain in maintaining stable signal output under sweat exposure, humidity, and variable loading conditions, as well as ensuring long-term mechanical durability and standardized performance evaluation across different systems.

In addition to motion sensing, TENG-based systems have been extensively explored for respiratory monitoring due to the continuous and periodic nature of breathing signals. Studies demonstrate that TENG-based respiratory monitoring systems can be broadly categorized into wearable textile-based sensors, airflow-driven devices, and hybrid self-powered systems [11,25,43,45,46,47,48]. Wearable TENG sensors integrated into masks, chest bands, and fabrics enable continuous monitoring of breathing patterns, including respiration rate, intensity, and airflow variations [11,47]. These systems have also been applied in sleep monitoring and apnea detection, providing non-invasive and real-time physiological data [48]. In addition, hybrid and multifunctional systems combine respiration sensing with other physiological signals, enhancing diagnostic capabilities and system reliability [25,45].

**Figure 6:**
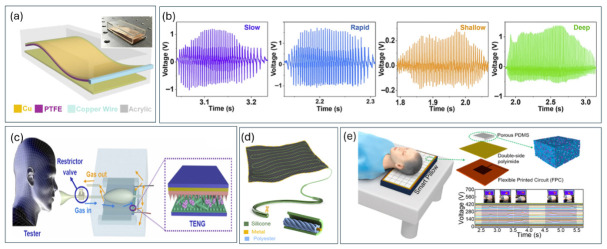
Respiration-driven TENG systems for healthcare monitoring: (**a**) aeroelastic vibration TENG design; (**b**,**c**) voltage output under different breathing patterns; (**d**) textile-based sleep monitoring system [47]; (**e**) intelligent pillow for head movement tracking [48].

Respiration is a continuous biomechanical process capable of generating substantial mechanical energy through thoracic expansion and diaphragmatic motion. As a key physiological indicator, respiratory rate and pattern provide critical insights into pulmonary and cardiovascular health. Contact-separation and single-electrode TENG configurations have been engineered to convert chest wall expansion and airflow variations into electrical signals for respiratory monitoring [47]. Respiration-driven TENG systems have demonstrated reliable detection of breathing patterns, including slow and deep respiration, as well as sleep monitoring applications [11,47,48]. Aeroelastic vibration-based TENGs (Figure 6a) demonstrate reliable detection of slow, rapid, shallow, and deep breathing patterns (Figure 6b,c), maintaining stable signal amplitude across varying intensities. Textile-based sleep-monitoring systems (Figure 6d) further enable wearable, washable platforms capable of detecting apnea-related events with high signal fidelity [47]. 

Beyond wearable systems, implantable respiration-driven TENGs have been proposed for monitoring lung expansion and diaphragm motion, with potential for chronic respiratory disease management. Intelligent pillow systems incorporating flexible TENG arrays (Figure 6e) additionally enable continuous sleep quality assessment through head movement tracking [47]. Despite high sensitivity and user comfort, challenges remain in maintaining long-term stability under humid conditions and repeated mechanical loading. A comparative summary of representative TENG-based respiratory monitoring systems, including wearable, airflow-driven, and hybrid configurations, is provided in Table 4.

TENG-based sensors have also demonstrated strong potential in monitoring muscle activity and body posture. TENG-based sensors have shown significant potential for monitoring muscle activity and body posture due to their ability to convert mechanical deformation into electrical signals. These systems are widely used in rehabilitation, prosthetic control, and human–machine interaction due to their flexibility, lightweight structure, and self-powered operation.

Recent studies demonstrate that TENG-based sensors have been widely applied for monitoring muscle activity and posture by converting biomechanical deformation into electrical outputs [25,43,46,49]. Wearable TENG systems integrated onto the skin or embedded in textiles enable real-time detection of muscle contraction, joint movement, and posture variations, making them suitable for rehabilitation monitoring and assistive technologies [44,46]. In addition, array-based TENG configurations improve spatial resolution and enable accurate pressure mapping for posture recognition and fall detection [50]. A comparative summary of representative TENG-based motion and strain sensing systems, including wearable, array-based, and hybrid configurations, is provided in Table 5.

**Figure 7:**
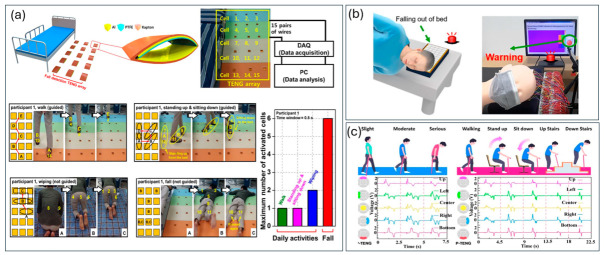
TENG-based posture and fall detection systems: (**a**) pressure-sensing TENG array; (**b**) wearable fall detection device; (**c**) AI-assisted mobility evaluation system [35].

Hybrid systems combining triboelectric and piezoelectric effects further enhance sensing sensitivity and stability under repeated mechanical loading [46]. Soft, stretchable TENG devices placed over muscle groups generate electrical outputs proportional to contraction intensity, supporting applications in rehabilitation, prosthetic control, and athletic monitoring [25,44,46,49]. A pressure-sensing TENG array consisting of arch-shaped thin-film units (Figure 7a) has been developed for fall detection and posture analysis, achieving high detection accuracy through spatial signal mapping [50]. Additional wearable designs include compact TENG sensors with rolling mechanisms for real-time fall detection, as well as AI-assisted smart walking sticks (Figure 7b,c) that integrate triboelectric sensing with machine learning for mobility assessment in elderly users. Furthermore, hybrid triboelectric–piezoelectric systems, such as PVDF-based nanocomposites and graphene-reinforced structures, enhance strain sensitivity and mechanical durability under cyclic loading, improving performance in wearable biomedical applications [36,46].

Despite these advancements, several challenges remain. Signal variability due to differences in sensor placement and user movement affects measurement consistency. In addition, the lack of standardized calibration methods limits quantitative interpretation of muscle activity signals. Long-term durability, mechanical fatigue, and user comfort also remain critical challenges for continuous wearable applications.

Overall, TENG-based physical sensing systems demonstrate high sensitivity and adaptability for wearable healthcare applications. However, several challenges remain, including signal instability under environmental conditions, motion artifacts, lack of standardized calibration, and long-term durability under continuous operation. Addressing these limitations is essential for translating these technologies into practical clinical applications.

### 5.2. Biochemical and Multifunctional Sensing

**Figure 8:**
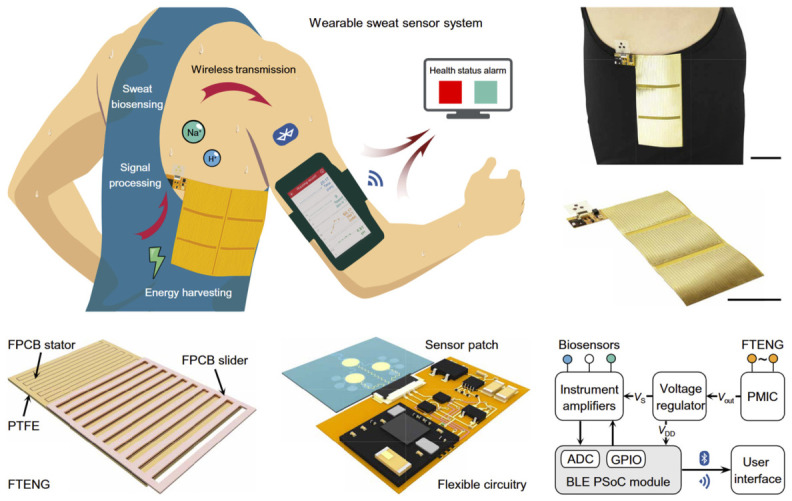
Integrated self-powered wearable system combining TENG-based energy harvesting, microfluidic sweat sensing, signal processing, and wireless data transmission for real-time health monitoring [36].

TENGs are increasingly being explored for biochemical sensing, expanding their role in personalized healthcare. By incorporating enzyme layers, hydrophilic polymers, and conductive composites, these systems can detect biomarkers such as glucose, electrolytes, and pH in biofluids [36]. Wearable platforms powered by biomechanical motion have demonstrated simultaneous energy generation and sweat analysis during physical activity [36]. Figure 8 illustrates a fully integrated, battery-free system combining energy harvesting, signal conditioning, microfluidic sweat sensing, and Bluetooth-based wireless transmission. Fabricated on a flexible printed circuit board (FPCB), the system conforms to the torso and employs a freestanding TENG (FTENG) with a grating slider and interdigital stator. It achieves a power density of 416 mW m^−2^ and supports real-time wireless data transmission during on-body testing [36]. This integration of energy harvesting, sensing, and communication represents a significant step toward autonomous, self-powered biosensing platforms. However, biochemical sensing with TENGs remains in an early stage of development, requiring improvements in sensitivity, selectivity, and long-term stability for clinical deployment.

## 6. TENGs for Cardiovascular Monitoring

Compared with general wearable sensing, cardiovascular monitoring demands higher signal fidelity, temporal resolution, and physiological accuracy, making it a key benchmark for evaluating TENG performance. The cardiovascular system produces continuous, periodic biomechanical signals through pulsatile blood flow, arterial wall expansion, and myocardial contraction. Recent studies, including those by Pullano et al. [11,48], have demonstrated triboelectric-based pseudo-impedance cardiography and wearable systems capable of clinically relevant hemodynamic monitoring. These approaches highlight the feasibility of TENG sensing for impedance-based cardiovascular assessment and risk evaluation. To enable cross-study comparison, Table 6 summarizes representative TENG-based cardiovascular monitoring systems, including sensing modality, anatomical placement, performance metrics, and limitations.

**Figure 9:**
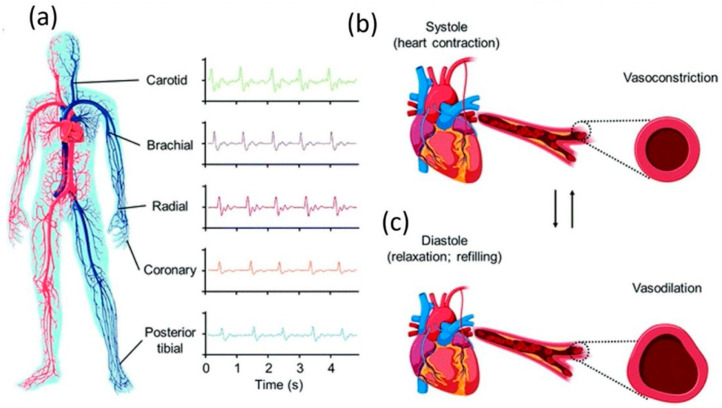
Arterial measurement sites and pulse-wave generation: (**a**) common non-invasive locations (carotid, brachial, radial, posterior tibial) with representative signals; (**b**) vasoconstriction during systole; (**c**) vasodilation during diastole [47].

As shown, significant variability exists in reported performance metrics, reflecting the lack of standardized validation protocols and the strong influence of device configuration, anatomical placement, and calibration strategy. These differences highlight the importance of physiological context in TENG-based sensing. Arterial pulse waves can be measured at multiple anatomical sites, including the carotid, brachial, radial, coronary, and posterior tibial arteries, where cyclic vasoconstriction during systole and vasodilation during diastole generate characteristic mechanical signatures (Figure 9) [43]. These low-frequency, highly periodic deformations are well suited for TENG-based sensing, enabling real-time extraction of pulse waveforms, heart rate, and derived cardiovascular parameters. Substantial progress has demonstrated the feasibility of cuffless blood pressure estimation and arterial stiffness assessment, supported by increasing in vivo and human validation studies [25,46].

Despite these advances, several challenges remain. TENG-based cardiovascular sensors are susceptible to signal drift during long-term operation, motion artifacts during daily activities, and variability across anatomical measurement sites. As a result, reported sensitivities and accuracy vary widely across studies, highlighting the need for standardized validation protocols and robust calibration strategies for clinical translation.

### 6.1. Pulse Waveform Extraction

High-fidelity pulse waveform acquisition forms the foundation of TENG-based cardiovascular sensing. When positioned over superficial arteries, periodic arterial wall displacement induces cyclic contact-separation, sliding, or electrostatic induction, generating voltage outputs proportional to local pressure fluctuations. Modern TENG devices can resolve fine waveform features, including the systolic peak, dicrotic notch, and diastolic reflection, which are critical for hemodynamic assessment. Flexible TENG patches placed at radial, carotid, and temporal sites have reconstructed pulse waveforms comparable to photoplethysmography (PPG) and arterial tonometry systems while maintaining self-powered operation [45].

For example, ridge-textured double-layer architectures enable simultaneous measurement of pulse pressure and interbeat intervals (IBI), capturing distinct systolic and diastolic morphology [27]. CNT/PDMS microcolumn-based TENGs further improve sensitivity to subtle arterial motion, producing IBI measurements consistent with ECG-derived signals [47]. Additionally, single-electrode and flexible thin-film configurations simplify device architecture while maintaining sufficient temporal resolution and Μv–mV sensitivity for detecting weak arterial pulses, supporting long-term wearable deployment [11]. These advances establish TENG-based pulse sensing as a robust, battery-free platform for continuous cardiovascular monitoring. Key material systems, device configurations, sensing sites, and performance metrics are summarized in Table 7, highlighting the relationship between structural design and sensing performance.

### 6.2. Heart-Rate Monitoring

**Figure 10:**
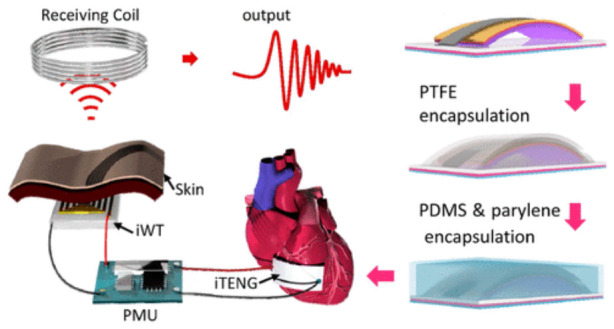
Schematic of a self-powered wireless cardiac monitoring system based on an implantable triboelectric nanogenerator (iTENG). The heartbeat-driven iTENG harvests biomechanical energy, which is regulated by a power management unit (PMU) and transmitted wirelessly via an implantable wireless transmitter (iWT) to an external receiving coil. Reproduced with permission from [57].

Beyond waveform reconstruction, TENGs enable extraction of heart rate and heart-rate variability (HRV) by converting periodic cardiac motion into electrical signals. Both wearable and implantable systems have been developed. Implantable TENGs (iTENGs) represent a major advancement. Zheng et al. demonstrated a heartbeat-driven iTENG in a large-animal model, achieving stable output and enabling wireless cardiac monitoring via a self-powered transmission system [48]. Zhao et al. further highlighted advantages including self-sustainability, flexibility, miniaturization, and biocompatibility [49]. As shown in Figure 10, iTENG-based systems integrate energy harvesting, power management, and wireless transmission. Multilayer encapsulation using PTFE, PDMS, and parylene ensures durability and insulation under physiological conditions. In this configuration, the iTENG functions simultaneously as an energy source and cardiac motion sensor.

### 6.3. Cuffless Blood Pressure Estimation

TENG-based systems enable cuffless blood pressure (BP) estimation using time-domain metrics such as pulse transit time (PTT), pulse arrival time (PAT), and pulse wave velocity (PWV). These metrics correlate inversely with systolic and diastolic BP when synchronized with ECG or dual-site measurements [50]. However, PTT- and PAT-based approaches are limited by dependence on individual calibration and variability in the pre-ejection period, introducing uncertainty under dynamic physiological conditions. Additionally, large-scale clinical validation against gold-standard methods remains limited. Flexible TENG patches enable PWV measurement with millisecond resolution, supporting real-time monitoring during rest and activity. Compared with oscillometric and PPG-based systems, TENG platforms offer continuous, self-powered BP tracking. Waveform morphology analysis further enables assessment of arterial stiffness through parameters such as augmentation index and reflection time. These features correlate with vascular elasticity and provide a low-cost alternative to tonometry and ultrasound-based systems.

Although cuffless blood pressure estimation using TENG-based sensors (e.g., PAT/PTT approaches) shows significant promise, several limitations remain. Most reported systems rely on subject-specific calibration, which limits their generalizability and long-term usability due to physiological variability. Furthermore, PAT includes the pre-ejection period (PEP), which varies with cardiac contractility and autonomic activity, introducing potential errors in BP estimation. As a result, PAT-based methods may not reliably reflect true vascular transit time. Pulse transit time (PTT)-based approaches partially mitigate this issue but still require calibration and are sensitive to measurement conditions and signal processing techniques. These factors should be carefully considered when comparing reported performances across studies.

### 6.4. In Vivo and Human Validation Studies

Translational progress has been supported by in vivo and human validation studies. Implantable TENGs have been evaluated in animal models for harvesting cardiac motion energy and powering microstimulators and pacemaker prototypes. Bioresorbable TENGs fabricated from biodegradable materials have demonstrated short-term cardiac pacing capability with controlled degradation. Human studies have shown reliable pulse and BP tracking using flexible epidermal TENG sensors under daily activity conditions, with strong agreement to clinical-grade systems. Across pulse waveform sensing, heart-rate monitoring, BP estimation, and arterial stiffness analysis, TENG-based systems demonstrate a unique combination of self-powered operation, mechanical flexibility, and multimodal sensing capability.

## 7. Implantable TENGs: Toward Self-Powered Cardiac and Internal Organ Interfaces

Unlike wearable platforms that harvest external motion, implantable TENGs directly convert biomechanical energy from internal organs into electrical output while simultaneously functioning as physiological sensors [56,58,59]. Advances in device miniaturization, mechanical compliance, and biocompatible and biodegradable materials have significantly improved feasibility for chronic implantation [56]. These developments position implantable TENGs at the intersection of energy autonomy and closed-loop therapeutic systems.

### 7.1. Heart-Wall Motion Harvesting

The rhythmic contraction and relaxation of the myocardium, along with pulsatile arterial expansion, provide continuous, high-frequency biomechanical energy. Flexible polymeric TENGs attached to the epicardial surface can convert cardiac motion into alternating electrical signals sufficient to charge capacitors or power low-energy electronics. Recent designs employ ultrathin, conformal membranes that reduce mechanical impedance and maximize interfacial contact with the moving heart wall. In small-animal models, such epicardial TENG systems demonstrated energy outputs capable of powering low-power pacemaker prototypes and telemetry modules. By harvesting energy during both systolic contraction and diastolic relaxation, these systems provide continuous electrical generation without reliance on electrochemical batteries. This approach directly addresses the finite lifespan and replacement risks associated with traditional implantable power sources. Despite promising demonstrations, mechanical coupling between device and myocardium must be carefully optimized to avoid restricting cardiac motion or inducing local tissue stress.

### 7.2. Myocardial Pressure and Hemodynamic Sensing

Beyond energy harvesting, implantable TENGs can function as real-time myocardial pressure and strain sensors. Because triboelectric output voltage is directly proportional to mechanical deformation, implanted devices can resolve systolic–diastolic transitions and intracardiac pressure fluctuations with high temporal resolution. Recent studies demonstrated that TENGs positioned on ventricular or atrial surfaces detect dynamic strain changes associated with cardiac contraction, enabling non-electrical measurement of hemodynamic parameters. Such systems offer potential for continuous, self-powered cardiac function monitoring and early detection of pathological changes, including ventricular hypertrophy or heart failure progression. Furthermore, integration with power management and wireless transmission modules enables closed-loop therapeutic systems in which implantable pacemakers or defibrillators could respond to TENG-derived physiological signals in real time. Ultrasound-driven hybrid TENG systems have further enhanced energy transduction efficiency and enabled wireless communication between implanted devices and external receivers, bridging the gap between invasive sensing and remote monitoring platforms.

### 7.3. Biocompatibility, Encapsulation, and Chronic Stability

While short-term in vivo studies demonstrate feasibility, long-term implantation introduces complex challenges. Conventional triboelectric materials such as PTFE and PDMS provide mechanical robustness and electrical performance but may trigger foreign-body response, fibrotic encapsulation, and protein adsorption that degrade signal quality over time. Chronic inflammation and encapsulation can alter mechanical coupling, reducing energy conversion efficiency. To mitigate these effects, multilayer encapsulation strategies incorporating parylene-C, silicone elastomers, or composite barrier coatings have been developed to provide electrical insulation, mechanical buffering, and reduced immune response. Surface passivation and hydrophobic coatings are being explored to minimize biofouling and maintain stable triboelectric output in wet, ionic environments. Maintaining consistent signal amplitude under continuous exposure to physiological fluids remains a key engineering challenge. Additionally, triboelectric output can be influenced by moisture, ionic conductivity, and tissue contact variability. Thus, long-term drift, signal reproducibility, and calibration reliability must be systematically evaluated before clinical deployment.

### 7.4. Biodegradable and Bioresorbable TENGs

To address issues associated with permanent implants and surgical removal, research has increasingly focused on biodegradable and bioresorbable TENGs (B-TENGs). These systems utilize transient polymers such as polylactic acid (PLA), polycaprolactone (PCL), silk fibroin, and gelatin, combined with biodegradable conductive materials such as magnesium or molybdenum. Such devices can generate stable electrical output for defined operational periods ranging from weeks to months before controlled bioresorption. Importantly, degradation byproducts including lactic acid and silk-derived peptides are biocompatible and metabolically manageable. Bioresorbable TENGs have demonstrated feasibility for short-term cardiac pacing, wound healing stimulation, and neural sensing applications. This emerging class of transient TENGs represents a paradigm shift toward temporary, self-powered implantable medical systems that reduce the need for secondary surgeries and minimize long-term foreign-body response.

Implantable TENGs uniquely integrate energy harvesting and physiological sensing within a single, battery-free architecture. Their ability to directly convert organ motion into electrical signals provides a compelling pathway for sustainable cardiac and internal organ monitoring. However, clinical translation requires addressing key challenges, including long-term biostability, encapsulation reliability, mechanical compatibility with dynamic tissues, and standardized performance validation. Establishing reproducible fabrication protocols and rigorous preclinical validation will be critical to advancing implantable TENGs from experimental systems to clinically viable technologies.

## 8. Hybrid and Intelligent TENG-Based Biomedical Systems

The evolution of TENGs from standalone energy harvesters to multifunctional biomedical platforms has been accelerated by integration with complementary sensing modalities, wireless electronics, and computational intelligence frameworks [55,56,57]. Hybrid architectures combining TENGs with conventional biosignal sensors, piezoelectric generators, low-power communication modules, and machine learning (ML) algorithms have demonstrated the feasibility of autonomous cardiovascular monitoring systems [48,51,57]. These platforms extend beyond energy harvesting, enabling multimodal signal acquisition, onboard processing, and real-time wireless transmission. This convergence represents a critical step toward closed-loop, intelligent, and personalized cardiovascular healthcare ecosystems [58,59].

Hybrid integration of TENGs with ECG and PPG sensors provides a powerful approach for comprehensive cardiovascular assessment. While TENGs capture mechanical pulse waveforms, ECG and PPG provide electrical and optical signals, respectively. Fusion of these modalities enables synchronized acquisition of cardiac events, supporting accurate derivation of PTT and PAT for cuffless blood pressure estimation and vascular stiffness analysis. Flexible epidermal platforms integrating TENG and ECG electrodes have demonstrated improved diagnostic accuracy and robustness compared to single-modality approaches. Multisensor fusion also reduces motion artifacts and enhances signal reliability under real-world conditions. However, calibration consistency across diverse physiological conditions remains a key challenge.

Consequently, hybrid piezoelectric-triboelectric (PENG-TENG) systems address limitations in energy density and output stability. PENGs generate output through intrinsic polarization under strain, complementing the transient, contact-based output of TENGs. This coupling improves energy conversion efficiency, broadens frequency response, and stabilizes output under dynamic physiological conditions. For example, ultrasound-driven hybrid systems have demonstrated efficient in vivo energy harvesting and wireless stimulation, while PVDF-graphene composites enable dual-mode sensing and energy generation. Despite performance gains, increased structural complexity and interfacial coupling challenges may affect long-term reliability and scalability.

Low-power wireless technologies, including Bluetooth Low Energy (BLE) and Near-Field Communication (NFC), have expanded the clinical utility of TENG-based platforms. BLE-enabled devices support continuous transmission of physiological data to mobile and cloud platforms, enabling remote monitoring and telemedicine applications. NFC-based architectures enable battery-free communication by directly coupling triboelectric energy to communication circuits. Integration with supercapacitors and power management circuits improves energy buffering and transmission stability. These platforms form a key component of the Internet of Medical Things (IoMT), supporting decentralized and long-term patient monitoring.

Integration of ML and artificial intelligence (AI) represents a major advancement toward intelligent diagnostics. TENG-derived signals contain rich morphological features that can be analyzed using time-frequency methods, support vector machines, and convolutional neural networks (CNNs). ML-assisted TENG platforms have demonstrated capabilities in arrhythmia detection, blood pressure estimation, and vascular stiffness analysis. Hybrid TENG–ECG systems enable automated anomaly detection and real-time hemodynamic modeling. However, clinical deployment requires large-scale datasets, cross-population validation, and improved model transparency.

The convergence of multimodal sensing, hybrid energy harvesting, wireless communication, and AI-driven analytics marks a transition from standalone TENG devices to fully autonomous cardiovascular systems. In these architectures, TENGs function simultaneously as energy sources, sensors, and triggers for therapeutic intervention. Future platforms may enable closed-loop systems in which TENG-derived physiological signals dynamically regulate pacemakers, drug delivery systems, or vascular assist devices. Realizing this vision will require advances in energy management, signal standardization, secure communication, and clinical validation.

## 9. Challenges and Current Research Gaps

Despite rapid advancements in TENG technology for wearable, cardiovascular, and implantable biomedical systems, significant scientific, engineering, and regulatory barriers remain before clinical translation can be achieved [60,61]. Between 2020 and 2025, substantial progress has been made in materials optimization, device engineering, and hybrid system integration; however, fundamental limitations in signal stability, long-term durability, standardization, and regulatory validation continue to restrict widespread medical deployment. Similar to early-stage electrochemical biosensors, the transition from proof-of-concept demonstrations to clinically robust devices will require rigorous validation, reproducibility, and alignment with regulatory standards.

A primary limitation of TENG-based wearable and implantable systems is signal instability under dynamic physiological and environmental conditions. Because triboelectric output depends on interfacial charge generation and retention, environmental humidity, perspiration, and biofluid exposure can induce charge leakage, dielectric screening, and voltage attenuation. In epidermal applications, sweat accumulation and variable skin contact significantly alter triboelectric performance, resulting in baseline drift and reduced signal fidelity. Motion artifacts further complicate physiological interpretation, particularly in cardiovascular monitoring, where subtle pulse waveform features must be resolved with high temporal precision. Strategies including hydrophobic surface coatings, ion-trapping nanolayers, and advanced signal filtering algorithms have partially mitigated these effects; however, stable operation under real-world conditions remains a significant challenge. Surface-engineered materials such as fluorinated polymers and graphene-based composites have demonstrated improved charge retention and environmental robustness. Nevertheless, standardized humidity tolerance metrics and long-term environmental stress testing protocols are still lacking.

Mechanical durability under continuous cyclic loading is a central concern, particularly for cardiovascular and implantable TENG systems subjected to millions to billions of deformation cycles. Repeated bending, compression, and shear stress may degrade triboelectric layers, leading to output decay and structural failure. Encapsulation strategies using parylene-C, PDMS, and silicone elastomers have improved resistance to biofluid infiltration and mechanical wear. However, encapsulation layers may stiffen, delaminate, or introduce mechanical impedance, thereby reducing sensitivity. Additionally, encapsulation can act as a triboelectric barrier, attenuating charge transfer efficiency. Emerging approaches focus on ultrathin, breathable, and conformal encapsulants that preserve mechanical compliance while preventing ionic infiltration. Achieving an optimal balance between electrical insulation, flexibility, and long-term adhesion under physiological strain remains a key materials engineering challenge.

Although TENG-based systems demonstrate strong potential for cuffless blood pressure estimation using PTT and PAT, clinical standardization remains unresolved. Inter-subject variability in vascular elasticity, arterial stiffness, and tissue composition introduces significant calibration challenges. Motion-induced timing errors and signal latency further complicate correlation between TENG-derived metrics and systolic/diastolic blood pressure values. Currently, no universally accepted calibration algorithms or validation frameworks exist for TENG-based BP monitoring. Therefore, large-scale population studies and standardized comparison with gold-standard oscillometric or invasive measurements are required for regulatory acceptance. Integration with multimodal sensing platforms, such as ECG/PPG fusion systems, may improve calibration robustness across diverse patient populations.

While short-term in vivo studies demonstrate feasibility of implantable TENG systems, long-term biological validation remains insufficient. Most experimental reports document operational stability over days to weeks, whereas clinical cardiac implants require multi-year reliability. Critical unknowns include chronic immune response, fibrotic encapsulation dynamics, mechanical coupling stability, and degradation pathways under sustained physiological exposure. Bioresorbable TENGs offer a promising strategy to mitigate long-term foreign-body response; however, controlled degradation kinetics are essential. Materials such as PLA and silk fibroin must maintain predictable electrical output during functional lifespan while degrading into non-toxic byproducts. Uncontrolled degradation could lead to performance fluctuation or premature failure. Hence, future work must incorporate chronic animal models, histological evaluation, and long-term biocompatibility assessments to validate safety and sustained functionality over clinically relevant timeframes.

Regulatory approval represents a substantial bottleneck in the clinical translation of TENG-based systems. Current frameworks from the U.S. Food and Drug Administration (FDA) and international regulatory bodies do not explicitly categorize self-powered or energy-harvesting medical devices, creating ambiguity in classification, testing standards, and risk assessment. Specific guidance on electrical safety thresholds, triboelectric charge exposure, electromagnetic compatibility, and long-term mechanical reliability is limited. For implantable applications, comprehensive evaluation of biocompatibility, encapsulation integrity, and electrical stability under physiological loading is required. For wearable systems, validation of long-term skin compatibility and data accuracy under real-world conditions is critical. Hybrid platforms integrating wireless transmission and AI-based analytics introduce additional regulatory considerations related to cybersecurity, data privacy, and algorithm transparency. Defining performance metrics specific to energy-harvesting medical devices will accelerate translation while ensuring patient safety. Establishing device-specific regulatory frameworks tailored to energy-harvesting biomedical systems will be critical for accelerating clinical translation while ensuring safety and performance consistency.

Overall, although TENG technology has advanced significantly in materials engineering, structural design, and system integration, its translational maturity remains limited. Bridging the gap between laboratory innovation and clinical deployment will require coordinated interdisciplinary efforts across materials science, biomedical engineering, clinical validation, and regulatory science. Future research should prioritize standardized durability testing under physiological conditions, large-scale human studies for validating cardiovascular accuracy, and the development of robust long-term encapsulation strategies. In addition, integrating multimodal sensing with artificial intelligence will enhance signal interpretation and reliability. Addressing these challenges will be critical for transforming TENG-based systems from promising experimental platforms into clinically viable, self-powered biomedical technologies.

## 10. Conclusions and Future Outlook

Over the past decade, particularly between 2020 and 2025, TENGs have evolved from fundamental demonstrations of contact electrification into sophisticated platforms for self-powered biomedical sensing and implantable healthcare systems. Advances in triboelectric materials, surface micro/nanostructuring, dielectric enhancement strategies, and flexible device architectures have significantly improved charge density, mechanical compliance, and operational stability under physiological motion. In parallel, system-level integration of power management, wireless communication, and multimodal sensing has transformed TENGs from passive energy harvesters into active, autonomous diagnostic systems. Contemporary platforms are now capable of harvesting biomechanical energy, resolving high-fidelity pulse waveforms, estimating cardiovascular parameters, and enabling wireless data transmission, establishing a foundation for battery-free, continuous health monitoring.

Despite this rapid progress, clinical translation remains constrained by persistent scientific and regulatory challenges. Signal instability under dynamic motion and humid biological environments continues to limit long-term reliability. Encapsulation strategies must balance mechanical compliance with electrical insulation to prevent performance degradation in chronic applications. Standardized calibration frameworks for blood pressure estimation and cardiovascular validation remain underdeveloped, limiting reproducibility across populations. Furthermore, long-term in vivo data for implantable systems, including chronic immune response and degradation kinetics of bioresorbable materials, are still insufficient. Addressing these challenges will require rigorous durability testing, large-scale human validation studies, and clearly defined regulatory pathways for self-powered medical devices.

The convergence of energy autonomy, intelligent signal processing, and biointegrated device design positions TENGs as a transformative technology for next-generation digital medicine. As TENG-based systems increasingly integrate with ML platforms and the IoMT, physiological motion may serve simultaneously as both an intrinsic power source and a clinically actionable signal. Future research should prioritize environmentally stable, biocompatible, and biodegradable materials capable of sustaining triboelectric performance under physiological conditions. At the systems level, deeper integration with piezoelectric harvesters, biochemical sensing modules, wireless telemetry, and AI-driven analytics will enable robust multimodal platforms for adaptive and personalized diagnostics. Equally important is the establishment of standardized clinical validation protocols aligned with FDA and ISO requirements to ensure measurement accuracy, safety, and reproducibility. Ultimately, the successful translation of TENG technology will depend on bridging materials innovation, device engineering, and clinical validation into unified, scalable systems capable of delivering reliable, self-powered healthcare solutions in real-world settings.

## Figures and Tables

**Table 1:** Quantitative comparison of TENG operating modes for biomedical applications.

Mode	Voltage (V)	Power Density (mW m^−2^)	Sensitivity	Complexity	Key Advantages	Limitations	Applications	Ref.
Contact-Separation	10–1000	up to 500	Very high (pressure)	Low	High output, simple design, strong signal	Mechanical fatigue, moisture sensitivity	Pulse, respiration, plantar pressure	[25,26,27]
Lateral Sliding	10–500	10–200	High (shear)	Moderate	Multidirectional motion sensing	Friction wear, durability issues	Joint motion, gait, rehab	[28,29]
Single-Electrode	1–100	1–50	Moderate	Very low	Easy integration with skin/textiles	Environmental dependence, lower output	Wearables, motion, pulse	[25]
Freestanding Layer	10–500	50–300	High	High	Efficient charge transfer, reduced wear	Complex fabrication, alignment challenges	Implantable, enclosed sensing	[30]

**Table 2:** Comparative analysis of triboelectric materials for biomedical TENG applications.

Material	Triboelectric/Behavior	Performance	Mechanical Properties	Biocompatibility	Advantages	Limitations	Applications	Ref.
PDMS	Moderate dielectric constant, negative polarity	Moderate	Highly flexible, stretchable	Good biocompatibility	Skin-conformal, stable under deformation	Low dielectric constant, requires modification	Pulse, respiration, wearable patches	[33]
PTFE, FEP	Strong negative polarity, high charge affinity	High voltage output, strong charge retention	Flexible, less stretchable than PDMS	Moderate biocompatibility	High output, stable signals	Limited stretchability	Motion, cardiovascular monitoring	[34,35]
CNT-PDMS, graphene, PVDF hybrids	Enhanced charge trapping, increased dielectric constant	High output, improved sensitivity	Flexible with improved durability	Composition-dependent	High sensitivity, improved SNR	Complex fabrication	Pulse, strain, hybrid sensing	[36]
PVA, polyacrylamide, ionic gels	High dielectric constant, ionic conductivity	Moderate-high output	Soft, stretchable	Excellent biocompatibility	Skin-like compliance, low impedance	Dehydration, instability	Wearable, implantable sensors	[37,38,39]
PLA, silk fibroin, gelatin	Moderate triboelectric performance	Moderate output	Flexible, tunable degradation	Excellent biocompatibility, biodegradable	Eco-friendly, no removal required	Limited long-term stability	Implantable, transient devices	[40]

**Table 3:** Comparison of TENG-based physical sensing systems for motion and strain detection.

Ref	Material/System	Output/Performance	Application	Key Advantage	Limitation
[48]	Textile-based TENG	50–200 V, sensitivity 0.1–1 V/kPa	Motion tracking	Flexible & breathable	Sweat sensitivity
[25]	Hydrogel-based TENG	10–100 V, high strain sensitivity (0.05–0.5 V/%)	Muscle sensing	Soft & conformable	Dehydration
[46]	Hybrid TENG/PENG	100–500 V, power density up to 300 mW/m^2^	Multi-motion sensing	Enhanced output	Complex design
[43]	PVDF-based hybrid	50–300 V, fast response (<50 ms)	Gait detection	Durable	Cost
[45]	Polymer TENG	100–400 V, sensitivity 1–10 V/N	Gesture recognition	High sensitivity	Wear fatigue
[25]	Wearable TENG	10–150 V, response time < 100 ms	Human motion monitoring	Lightweight	Calibration
[31]	Biophysical TENG	20–200 V, multi-signal detection	Physiological sensing	Multifunctional	Signal noise
[37]	Hydrogel bioelectronics	5–50 V, low-pressure detection (<10 Pa)	Soft tissue motion	Biocompatible	Stability
[38]	Hydrogel TENG	10–120 V, moderate sensitivity	Sports monitoring	Stretchable	Mechanical fatigue
[7]	Nanocomposite TENG	100–500 V, enhanced efficiency	Strain sensing	Improved output	Fabrication complexity

**Table 4:** Comparative summary of TENG-based respiratory monitoring systems.

System Type	Configuration	Output (Typical)	Application	Advantage	Limitation	Ref
Respiration-driven TENG	Airflow-based	5–50 V	Breathing monitoring	Self-powered	Low output	[47]
Wearable TENG	Textile/chest band	10–100 V	Continuous respiration	Comfortable	Motion artifacts	[11]
Sleep monitoring TENG	Wearable	Stable signal	Apnea detection	Non-invasive	Humidity sensitivity	[48]
Wearable sensor	Flexible	10–150 V	Multi-physiological &	Lightweight	Calibration	[25]
Biophysical TENG	Hybrid	20–200 V	Respiration motion	Multi-sensing	Noise	[46]
Biomaterial TENG	Wearable	Moderate	Clinical monitoring	Biocompatible	Stability	[43]
Self-powered biosensor	Integrated	Moderate	Physiological sensing	Autonomous	Early-stage	[45]

**Table 5:** Comparison of recent triboelectric nanogenerator (TENG)-based motion and strain sensors.

System	Application	Advantage	Ref
Wearable TENG	Muscle sensing	Flexible	[25]
Array TENG	Posture detection	High accuracy	[50]
Hybrid system	Multi-sensing	High sensitivity	[46]

**Table 6:** Quantitative comparison of TENG-based cardiovascular monitoring systems.

Device Type	Anatomical Site	Signal	Sensitivity	BP Error	Stability	Key Condition	Limitation	Ref.
Pulse TENG	Radial artery	Pulse wave	Qualitative (high)	±5–10 mmHg	Moderate	Human, resting	Motion artifacts	[31]
PTT-based	Wrist & chest	BP estimation	Moderate	±5 mmHg	Low drift	Human, calibrated	Calibration needed	[51]
Implantable	Cardiac	Pressure	High	NA	High	Animal/in vivo	Invasive	[51]

**Table 7:** TENG-based pulse waveform extraction for cardiovascular monitoring.

Tribo-Materials/Electrodes	Configuration	Site	Output/Sensitivity	Measurement Context	Key Result	Ref.
TPU-ridge elastomer	Double-layer TENG	Radial	490 mW m^−2^	Controlled lab	Pulse & HR monitoring	[27]
PTFE; Cu/Au	Wearable TENG	Wrist, carotid	mV–V; SNR 45 dB	Human	Clear pulse waveform	[52]
PDMS (tribo-layer); ITO/PET (electrode)	Single-electrode TENG (trench-structured flexible sensor)	Radial artery (wrist)	0.3–1 nA current (amplified signal)	Human pulse monitoring (wearable)	Clear pulse waveform (incident and reflected waves); AIr ≈ 0.51; ΔTDVP ≈ 0.27 s	[53]
PDMS ion gel/PVDF-HFP nanofiber; Cu; Kapton	Contact-separation TENG	Wrist (radial)	0.43 kPa^−1^; 0.068–0.102 V·kPa^−1^; 31 V	Pressure & pulse sensing	Wrist pulse detected; wide range (0.01–700 kPa); 0.9 W/m^2^	[54]
PVDF nanofiber/TPU nanofiber; MWCNT electrode	Single-electrode stretchable TENG	Finger, hand motion	218 V; ~4.5 µA; 135 mW	Wearable motion sensing	Detects finger motion (bending, clapping, grabbing); stable under deformation	[55]
PDMS/VHB elastomer; PAAm–LiCl hydrogel (ionic electrode); Al/Cu contact	Single-electrode STENG (hydrogel-based stretchable TENG)	Human skin (hand, touch)	145 V; 47 nC; 35 mW m^−2^	Biomechanical energy harvesting & tactile sensing	Detects touch/pressure; powers LEDs & wearable devices; highly stretchable.	[56]

## Data Availability

No new data were created or analyzed in this study. Data sharing is not applicable to this article.
